# Synthesis of CdIn_**2**_S_**4**_ Microsphere and Its Photocatalytic Activity for Azo Dye Degradation

**DOI:** 10.1155/2014/241234

**Published:** 2014-03-30

**Authors:** Jianhui Huang, Wei Lin, Jianqin Chen

**Affiliations:** ^1^College of Environmental & Biological Engineering, Putian University, Putian 35110, China; ^2^Department of General Surgery, Affiliated Hospital of Putian University, Putian 351100, China

## Abstract

CdIn_2_S_4_ was prepared by ultrasonic spray pyrolysis. The prepared CdIn_2_S_4_ was characterized by X-ray diffraction (XRD), field emission scanning electron microscopy (FSEM), transmission electron microscopy (TEM), UV-visible diffuse reflectance spectroscopy (UV-Vis DRS), and N_2_-sorption techniques. Aqueous photocatalytic activity was evaluated by the decomposition of methyl orange under visible light irradiation. The results indicate that the prepared CdIn_2_S_4_ has spherical morphology with mesoporous structure which can efficiently degrade methyl orange in water. The sample prepared at 500°C exhibits the optimized photocatalytic activity.

## 1. Introduction

Azo dyes, a large class of synthetic organic dyes that contain nitrogen as the azo group –N = N– as part of their molecular structures. More than half the commercial dyes belong to this class which have been widely used in many industries such as the production of textiles, paint, ink, and cosmetics [1]. The effluent from azo dyes industries contains substantial amounts of such dyes and their breakdown products, which causes coloration of water and poses a threat to aquatic life [2–4], mutagenic/carcinogenic [5], and genotoxic [6]. Therefore dyes effluent poses a great threat to the environment and increasing attention has been paid to the treatment of dye effluent. While due to the complex aromatic structure and stability of these dyes, conventional biological treatment processes are ineffective for their degradation [7]. The absorption and coagulation processes are only able to absorb them up to some extent, but the sludge produced from both processes becomes more difficult to be disposal [8].

In the past several decades, semiconductor-mediated photocatalytic oxidation has been proved to be a promising technology for the treatment of water containing azo dyes [9]. Among a variety of semiconductor materials, the titanium dioxide (TiO_2_) photocatalysts have been most extensively investigated and used because of their physical properties and economic advantages, such as high redox power, photo-stability, chemical inertness, and cheapness [10]. However, TiO_2_ can only absorb a very small ultraviolet part (3-4%) of solar light because of its wide band gap (3.2 eV for anatase) which constrains the extensive applications of TiO_2_. Thus, considerable efforts have been devoted to develop new visible responded photocatalyst which is still a significant issue in both academic and practical applications.

Among various new photocatalysts, multinary chalcogenide semiconductors have attracted great attention because of the instability of the mono- and binary chalcogenide compounds [11–14]. Cadmium indium sulphide (CdIn_2_S_4_) belonging to ternary semiconductors of chalcogenide AB_2_X_4_ and being considered to have potential applications in optical devices, solar cells [15] and photocatalysts for H_2_ evolution, bacterial inactivation, and organic pollutant elimination [16–18]. Considering the advantages of nanostructures over the bulk materials, attempts have been made to synthesize CdIn_2_S_4_ with variety of nanostructures. Up to now, the nanostructural CdIn_2_S_4_ has been synthesized by methods like hydrothermal [15, 19], microwave [20], and sol-gel [21] with template. In most cases, these techniques require high temperature or high pressure to obtain crystalline material. In addition, the removal of the templates requires additional processing steps that can be costly and wasteful. Thus, a high-yield synthetic and template-free method for the CdIn_2_S_4_ preparation can undoubtedly benefit the design of high-performance materials in catalytic applications. Recently, ultrasonic spray pyrolysis technique (USP) is particularly attractive. It is fast, inexpensive, vacuumless, and suitable for mass production [22–24]. In the previous work, we have demonstrated that the USP method is a common method to prepare multinary sulfide [25].

In present work, the CdIn_2_S_4_ microspheres were synthesized using USP process. The effect of reaction conditions on the prepared CdIn_2_S_4_ was studied. The obtained microspherical CdIn_2_S_4_ was used as photocatalyst to oxidize azo dyes in aqueous solution.

## 2. Experimental Section

### 2.1. Catalysts Preparation

All of the reagents were of analytical grade and used without further purification. The CdIn_2_S_4_ photocatalyst was prepared by a USP method. In a typical synthesis, 0.532 g Cd (AC)_2_ *·*2(H_2_O), 1.2 g InCl_3_(H_2_O)_4_, and 0.76 g CH_4_N_2_S were added into 150 mL water under magnetic stirring to form a transparent solution. The solution was nebulized at 1.7 MHz ± 10% (YUYUE402AI, Shanghai) and then carried by air with a flow rate 10 L/min through a quartz tube surrounded by a furnace thermostated at 500~700°C. The quartz reaction tube with the diameter of 3.5 cm was 1 m long. The products were collected in a percolator with distilled water, separated by centrifugation, and washed thoroughly with ethanol and distilled water. The product was finally dried in an oven at 60°C for overnight.

### 2.2. Characterization

Powder X-ray diffraction (XRD) patterns were collected in *θ*-*θ* mode using a Bruker D8 Advance X-ray diffractometer (Cu K*α*1 irradiation, *λ* = 1.5406 Å). The morphology of the sample was investigated by field emission scanning electron microscopy (FSEM) (JSM-6700F). Transmission electron microscopy (TEM) was recorded on a FEI Tecnai 20 microscope. Carbon-coated copper grid was used as the sample holder. The sample was suspended in ethanol solution, followed by sonication for 30 min in the ultrasonic bath. Drops of the suspensions were applied on a copper grid coated with carbon. Nitrogen sorption experiments were carried out at 77 K by using Micromeritics ASAP 2020 equipment. All the samples were degassed at 140°C and 10^−6^  torr for 6 h prior to the measurement. The UV-visible diffuse reflectance spectra were performed on a Varian Cary 500 spectrometer with an integrating sphere attachment ranging from 200 nm to 800 nm. BaSO_4_ was used as a reference sample in these measurements.

### 2.3. Evaluation of Photocatalytic Activity

The photocatalytic activities of the samples were evaluated by the decomposition of methyl orange (MO) a typical azo dye in aqueous solution. Catalyst (0.05 g) was suspended in a 100 mL Pyrex glass vessel containing MO with a concentration of 25 ppm. The visible light source was a 300 W halogen lamp (Philips Electronics) positioned beside a cylindrical reaction vessel with a flat side. The system was water-cooled to maintain the temperature. A 400 nm cutoff filter was placed in front of the vessel to ensure irradiation by visible light. The suspension was stirred in darkness for 1 h to achieve adsorption equilibrium and the reactor was irradiated to induce photocatalyzed decomposition reactions. At given irradiation time intervals, 3 mL of the reaction suspension was collected and centrifuged to remove the catalyst. The degraded solution was analyzed using a Varian Cary 50 Scan UV-Vis spectrophotometer.

## 3. Results and Discussion

### 3.1. Crystal Structure

**Figure 1:**
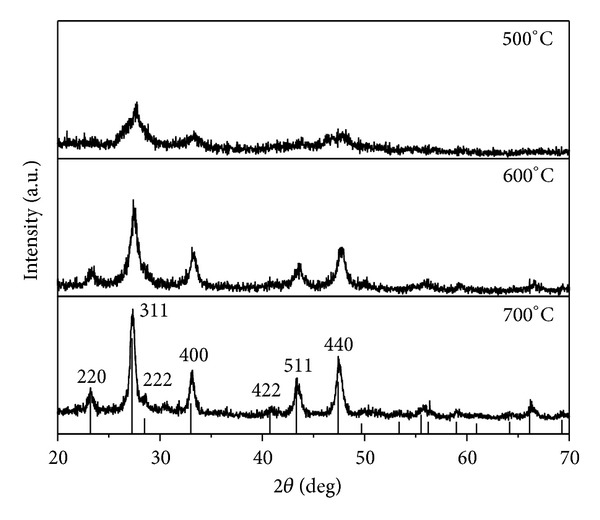
XRD patterns of CdIn_2_S_4_ prepared at different temperatures.

The X-ray diffraction (XRD) patterns of the resultant products synthesized from USP process at different temperature are shown in Figure 1. The XRD patterns of the sample prepared at 700°C can be indexed to cubic CdIn_2_S_4_ well consistent with the reported data (JCPDS number 27-60, space group: Fd-3 m). The peaks at 2*θ* values of 23.18, 27.25, 28.48, 33.00, 40.74, 43.31, and 47.40 match well with the (220), (311), (222), (400), (422), (511), and (440) crystal planes of CdIn_2_S_4_, respectively. There is no any trace of impurity phase such as CdS, In_2_S_3_ under the instrument resolution, indicating the high purity of the sample. Furthermore, the intensity of the peaks according to CdIn_2_S_4_ is affected by the preparation temperature. The XRD pattern for the sample prepared at 500°C shows broad noisy peaks means the semi crystalline nature of the sample. The intensity of peaks is increasing with the increasing of temperature. This suggests that the crystalline of CdIn_2_S_4_ increases with the increase of temperature. The calculated average crystal sizes of the samples prepared at 500, 600, and 700°C using the Scherrer equation are 5.4, 8.4, and 14.2 nm, respectively.

### 3.2. Morphology

**Figure 2:**
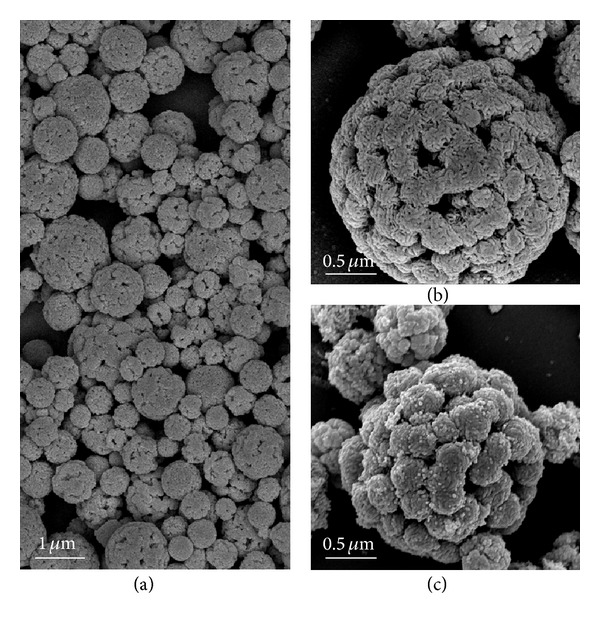
The FSEM images of CdIn_2_S_4_ prepared by ultrasonic spray pyrolysis. (a) and (b) 500°C, (c) 700°C.

Figure 2 depicts the FSEM images of prepared samples. Figure 2(a) is the low-magnification image of the sample prepared at 500°C which indicates that the as-prepared samples consist entirely of spheres with a range of 100 nm to 1.5 *μ*m. The ultrasonic nebulizer during the preparation is the key to form the spherical morphology. The aerosol produced from nebulizer consisted of numerous small liquid droplets which contained the precursor. These droplets would serve as microreactors and yield one spherical particle per droplet. The size of droplet and concentration of precursor determines the product dimension [26]. When these droplets are carried into the tubular reactor with air, the water in the droplet is heated and evaporating. At the same time, the thiourea in the droplet would decompose quickly and release H_2_S which would react with the metal precursor quickly and generate submicrospherical CdIn_2_S_4_. The excess H_2_S from thiourea will be absorbed by the water in the percolator which will not be released out of system. Figure 2(b) is the magnified surface morphologies of samples prepared at 500°C which shows the porous structure of sample. The porous structure will not be destroyed even prepared at high temperature of 700°C (see Figure 2(c)). The result also shows that the sample prepared at 500°C has a crude surface compared with the sample prepared at 700°C. This can be due to the larger catalyst particles for the sample prepared under higher temperature.

**Figure 3:**
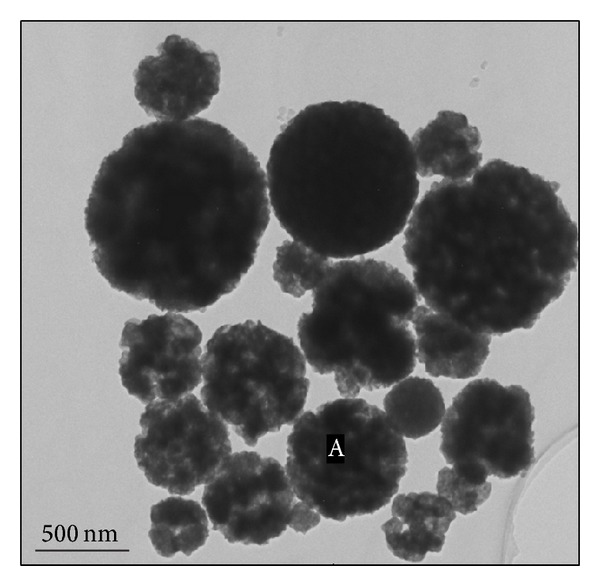
The TEM images of CdIn_2_S_4_ prepared at 500°C.

**Figure 4:**
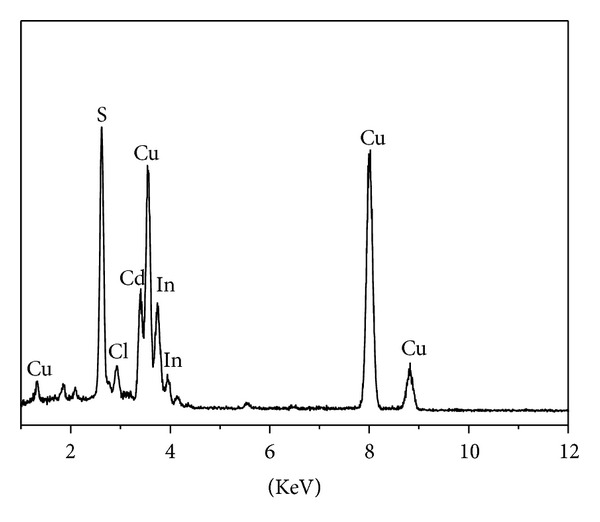
Energy-dispersive X-ray analysis spectra of CdIn_2_S_4_ prepared at 500°C.

The TEM image of CdIn_2_S_4_ sphere in Figure 3 confirms that the prepared spheres are composed of small nanoparticles and have porous structure. The porous structure will endow the CdIn_2_S_4_ with more active points and larger light-harvesting ability which will benefit its photocatalytic performance. The composition of CdIn_2_S_4_ sphere was determined by energy dispersive X-ray spectroscopy (EDX) in the “A” position of Figure 3. The results of EDX analysis (Figure 4) imply that the as-prepared samples contain In, Cd, S, and a small amount of Cl element. The ratio of In to Cd in the microspheres is 1.95 which is very close to the atom ratio in CdIn_2_S_4_. The small amount Cl element is from the precursor of InCl_3_(H_2_O)_4_ which is not completely removed during the washing of product.

### 3.3. Optical Properties

**Figure 5:**
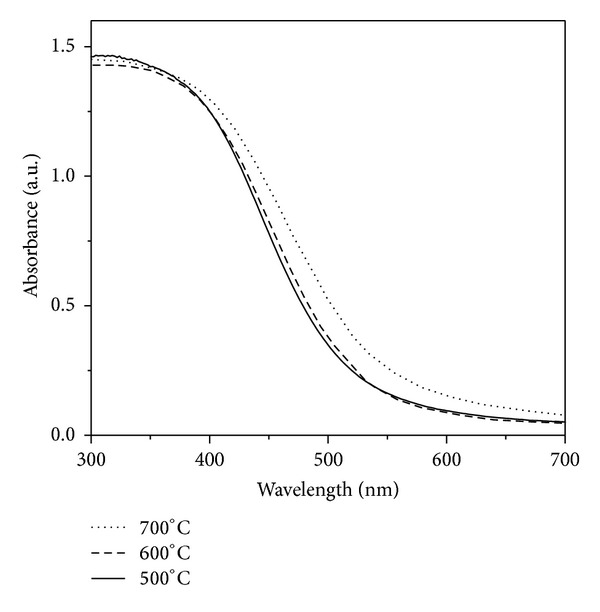
Diffuse reflectance UV-Vis spectra of CdIn_2_S_4_ prepared at different temperature.

Figure 5 shows the light absorption properties of prepared samples. All the samples show remarkable absorption in the visible light region. Besides, the basic adsorption edge of the CdIn_2_S_4_ is shifted to shorter wavelength with the decreasing of temperature in preparation. The absorption edges of CdIn_2_S_4_ prepared at 500, 600, and 700°C are 522, 528, and 541 nm corresponding to a band gap of 2.38, 2.35, and 2.29 eV, respectively. The blue shifts of adsorption edge are due to the quantum confinement effect, which is attributed to the smaller crystal size of CdIn_2_S_4_ prepared in low temperature.

### 3.4. Specific Surface Area and Pore Structure

**Figure 6:**
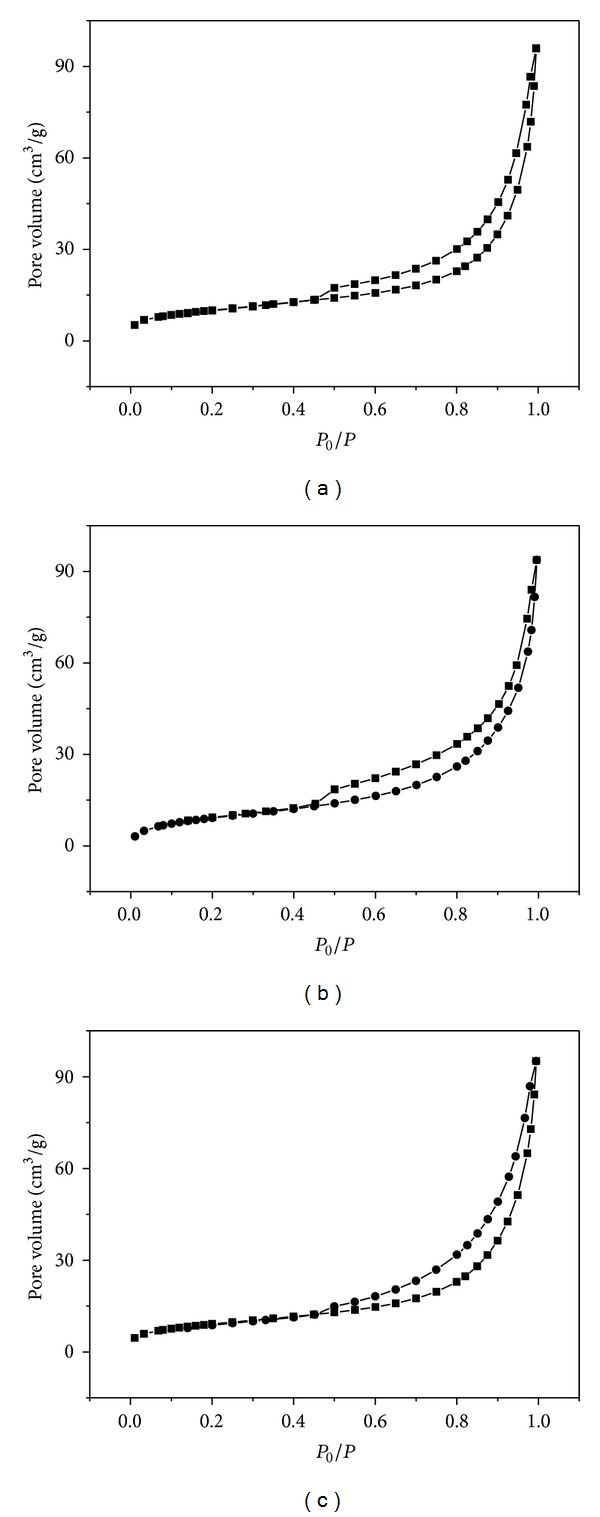
N_2_ adsorption-desorption isothermal curves of CdIn_2_S_4_ prepared at different temperatures (a) 500°C, (b) 600°C, and (c) 700°C.

Figure 6 shows the nitrogen adsorption-desorption isotherms of the CdIn_2_S_4_ prepared at different temperature. All the samples show similar type-IV isotherms, which are representative of mesoporous solids. In addition, the surface area, pore size, and pore volume (see Table 1) have no significant difference for the samples prepared in the different temperature. This indicates that the mesoporous structures was not destroyed or did not shrink in the high temperature which is consistent with the result of SEM in Figure 2.

### 3.5. Photocatalytic Activity

**Figure 7:**
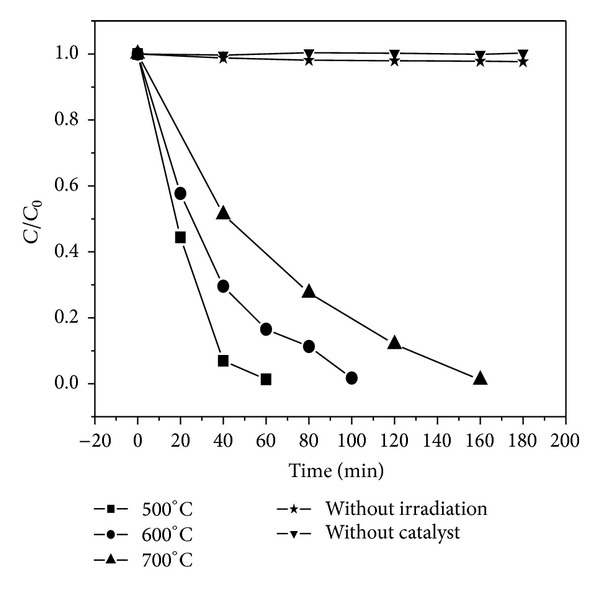
Photocatalytic activity of the CdIn_2_S_4_ sphere samples.

Figure 7 shows the photocatalytic degradation of methyl orange (MO) on CdIn_2_S_4_ microspheres with otherwise identical conditions under visible-light irradiation (*λ* > 400 nm) after the adsorption desorption equilibrium was reached. The control test (without catalyst) under visible light irradiation showed that the photolysis of MO was negligible. The dark control experiment with CdIn_2_S_4_ prepared at 500°C alone reveals that the concentration of MO was also unchanged after 3 hours, which demonstrates that the adsorption/desorption equilibrium had been obtained after 1 hour in dark and the CdIn_2_S_4_ microspheres would not react with MO. The MO was degraded quickly in the presence of CdIn_2_S_4_ under visible light irradiation and indicates the high photocatalytic activity of CdIn_2_S_4_ microspheres. The test also shows that the activities of doped samples were greatly influenced by the prepared temperature. The complete degradation of methyl orange for samples prepared at 500, 600, and 700°C needs 60, 100, and 160 min, respectively. Considering the similar surface area and pore structure among different samples, the higher photocatalytic activity for the sample prepared at 500°C can be due to the small crystal size which is of benefit to the separation of excited electron/hole pair during photocatalytic reaction.

## 4. Conclusions

The CdIn_2_S_4_ have been synthesized by using ultrasonic spray pyrolysis process without using any template. The prepared samples have spherical morphology with mesoporous structure whose products can be generated continuously at a rate of several grams per hour. The prepared CdIn_2_S_4_ exhibited excellent photocatalytic activity. We expect that these CdIn_2_S_4_ spheres prepared with this method will have immense importance in other regions such as solar cells, LED, and optoelectronics.

## Figures and Tables

**Table 1:** Crystalline and porous properties of CdIn_2_S_4_ prepared at different temperatures.

Temperature (°C)	*d* ^a^ (nm)	Pore size^b^ (nm)	Surface area^c^ (m^2^g^−1^)	Pore volume^d^ (cm^3^g^−1^)
500	5.4	13.5	36.1	0.15
600	8.4	13.5	34.5	0.15
700	14.2	13.6	33.5	0.15

^a^ *d* values of the samples estimated by Scherrer formula; ^b^BJH pore sizes determined from the nitrogen desorption branches; ^c^BET surface areas determined from the nitrogen adsorption and desorption isotherm measurement; ^d^pore volumes is the BJH desorption cumulative pore volume of pores.
